# CLUE: a randomized comparative effectiveness trial of IV nicardipine versus labetalol use in the emergency department

**DOI:** 10.1186/cc10289

**Published:** 2011-06-27

**Authors:** W Frank Peacock, Joseph Varon, Brigitte M Baumann, Pierre Borczuk, Chad M Cannon, Abhinav Chandra, David M Cline, Deborah Diercks, Brian Hiestand, A Hsu, Preeti Jois-Bilowich, Brian Kaminski, Philip Levy, Richard M Nowak, Jon W Schrock

**Affiliations:** 1 Department of Emergency Medicine, The Cleveland Clinic, 9500 Euclid Avenue, Cleveland, OH, 44195 USA; 2 The University of Texas Health Science Center at Houston and The University of Texas Medical Branch at Galveston, Galveston, Texas, 77555 USA; 3 Division of Clinical Research and Associate Professor of Emergency Medicine, Cooper University Hospital, Camden, NJ, 08103-1489 USA; 4 Department of Emergency Medicine, Massachusetts General Hospital, Boston, MA, 02114 USA; 5 Department of Emergency Medicine, University of Kansas Hospital, Kansas City, KS, 66103-2918 USA; 6 Division of Emergency Medicine, Duke University Medical Center, Durham, NC, 27710 USA; 7 Wake Forest University School of Medicine, Winston Salem, NC, 27157 USA; 8 University of California, Davis Medical Center, Sacramento, CA, 95817 USA; 9 The Ohio State University, Columbus, OH 43210 USA. Currently affiliated with the Department of Emergency Medicine, Wake Forest University Health Sciences, Winston-Salem, North Carolina, 27157 USA; 10 The Cleveland Clinic, 9500 Euclid Avenue,Cleveland OH, 44195 USA; 11 Department of Emergency Medicine, University of Florida College of Medicine, Gainesville FL, 32610-0277 USA; 12 Toledo Hospital, Toledo OH, 43606 USA; 13 Department of Emergency Medicine, Wayne State University School of Medicine and the Cardiovascular Research Institute, Wayne State University (both in Detroit, MI), 48201-1998 USA; 14 Department of Emergency Medicine, Wayne State University School of Medicine, Henry Ford Hospital, Detroit, Michigan, 48202-2689 USA; 15 Department of Emergency Medicine, MetroHealth Medical Center, Case Western Reserve University School of Medicine, Cleveland, OH 44109 USA

## Abstract

**Introduction:**

Our purpose was to compare the safety and efficacy of food and drug administration (FDA) recommended dosing of IV nicardipine versus IV labetalol for the management of acute hypertension.

**Methods:**

Multicenter randomized clinical trial. Eligible patients had 2 systolic blood pressure (SBP) measures ≥180 mmHg and no contraindications to nicardipine or labetalol. Before randomization, the physician specified a target SBP ± 20 mmHg (the target range: TR). The primary endpoint was the percent of subjects meeting TR during the initial 30 minutes of treatment.

**Results:**

Of 226 randomized patients, 110 received nicardipine and 116 labetalol. End organ damage preceded treatment in 143 (63.3%); 71 nicardipine and 72 labetalol patients. Median initial SBP was 212.5 (IQR 197, 230) and 212 mmHg (IQR 200,225) for nicardipine and labetalol patients (*P *= 0.68), respectively. Within 30 minutes, nicardipine patients more often reached TR than labetalol (91.7 vs. 82.5%, *P *= 0.039). Of 6 BP measures (taken every 5 minutes) during the study period, nicardipine patients had higher rates of five and six instances within TR than labetalol (47.3% vs. 32.8%, *P *= 0.026). Rescue medication need did not differ between nicardipine and labetalol (15.5 vs. 22.4%, *P *= 0.183). Labetalol patients had slower heart rates at all time points (*P *< 0.01). Multivariable modeling showed nicardipine patients were more likely in TR than labetalol patients at 30 minutes (OR 2.73, *P *= 0.028; C stat for model = 0.72)

**Conclusions:**

Patients treated with nicardipine are more likely to reach the physician-specified SBP target range within 30 minutes than those treated with labetalol.

**Trial registration:**

ClinicalTrials.gov: NCT00765648

## Introduction

Hypertensive emergencies require immediate, controlled blood pressure (BP) reduction and intensive care monitoring to avoid or limit end-organ damage [1]. Without proper treatment, the one year mortality of hypertensive emergencies is as high as 79%. With appropriate treatment, this decreases to 25% [2]. Although BP reduction is essential, antihypertensive therapy must be tailored to each patient's specific needs and clinicians must avoid the potential for harm caused by excessive BP lowering [1,3]. Too great or too fast of a reduction in BP may lead to end-organ hypoperfusion, potentially resulting in ischemia and infarction [1]. Unfortunately, a lack of acute clinical trials has left clinicians with little evidence-based guidance as to the optimal agent for BP control. Two agents commonly used for the management of acute hypertensive crises are intravenous (IV) nicardipine and labetalol. Nicardipine is a titratable IV dihydropyridine calcium ion influx inhibitor (i.e., calcium channel blocker) with dosing that is independent of body weight. It is given as an infusion and its onset of action is 5 to 15 minutes, with a clinical offset of activity (defined as a 10 mmHg increase in systolic blood pressure (SBP) or diastolic blood pressure (DBP) after stopping infusion) within 30 minutes (range of 5 to 120 minutes) [4]. After an IV infusion, nicardipine plasma concentrations decline tri-exponentially, with a rapid early distribution phase (α-half-life of 2.7 minutes), an intermediate phase (β-half-life of 44.8 minutes), and a slow terminal phase (γ-half-life of 14.4 hours) that can only be detected after long-term infusions. Nicardipine is rapidly and extensively metabolized by the liver, with excretion roughly equally in the feces and urine. Although nicardipine is as effective as sodium nitroprusside at lowering SBP, unlike nitroprusside, nicardipine reduces both cardiac and cerebral ischemia [4]. Nicardipine has high arterial vascular selectivity, with strong coronary and cerebral vasodilator effect that results in increased coronary and cerebral blood flow [5].

Labetalol hydrochloride is an IV antihypertensive with both selective alpha- and non-selective beta-adrenergic receptor blocking actions. Labetalol is recommended to be given as a bolus injection, with dose escalations every 10 minutes until the goal BP is reached. Metabolized by the liver to form an inactive glucuronide conjugate, it has an onset of action within two to five minutes, reaches peak effects at 5 to 15 minutes, has an elimination half life of 5.5 hours, and duration of action of up to four hours.

In a recent retrospective analysis of neurologic critical care cerebrovascular accident (CVA) patients, nicardipine required fewer dosage adjustments than labetalol, and provided decreased need for additional use of antihypertensives agents [6]. It is unknown whether these findings would translate to other patient populations in other care settings. Thus far, no emergency department (ED) comparative effectiveness trial of these agents has been conducted. Our purpose was to perform a phase four, randomized, comparative effectiveness trial to determine the efficacy and safety of a premixed nicardipine infusion versus IV bolus labetalol for management of hypertension in the ED setting.

## Materials and methods

This investigation was performed at 13 US academic EDs, each with institutional review board approval. Registered at ClinicalTrials.gov, with identifier is NCT00765648, this study was conducted in accordance with Good Clinical Practices and in compliance with all applicable subject privacy requirements.

After meeting all eligibility criteria, and obtaining consent, patients were prospectively enrolled into the study. Before randomization, which was stratified by center, the treating physician was required to define a target SBP. The target SBP was determined at the discretion of the physician, based on their impression of necessity for a given clinical scenario. A target range was defined as the target SBP ± 20 mmHg. To meet the primary endpoint, patients were required to be within the target SBP range by 30 minutes; transient time but not within the target range at 30 minutes was not considered to be within the target range. Subjects were then randomized in a 1:1 ratio to receive either a nicardipine infusion as a premixed formulation or bolus IV labetalol. The active treatment phase was 30 minutes. Any treatment after 30 minutes was at physician discretion. The study data collection period was for the first six hours following enrollment.

To be eligible for CLUE, patients had to be older than18 years of age, with a SBP of 180 mmHg or more on two consecutive readings (10 minutes apart), and able to provide signed informed consent, including authorization to use protected health information. Patients were ineligible if they had specific contraindications to receiving either a beta blocker or a calcium channel blocker, or if they were believed to suffer from a condition associated with an evidence-based guideline indication precluding randomization to another agent (e.g., in the setting of an acute myocardial infarction, beta-blockade is indicated and patients were excluded as they should not be randomized to a calcium channel blocker). Patients were also excluded if they met any of the following criteria: use of any investigational drug within 30 days, pregnant or breast-feeding, contraindications or allergy to beta-blockers or calcium channel blockers, advanced aortic stenosis, bronchial asthma, overt cardiac failure, greater than first-degree heart block, cardiogenic shock, severe bradycardia, obstructive airway disease, decompensated heart failure, a known left ventricular ejection fraction of less than 35%, history of CVA within 30 days, known impaired hepatic function, suspected myocardial infarction, suspected aortic dissection, suspected cocaine use as the cause of ED presentation, or if they were concurrently receiving any IV antihypertensive medication.

Although medication dosing was per the physician's discretion, the US Food and Drug Administration (FDA) recommended dosing schedules were provided, and their use encouraged. We chose the FDA recommendations because this study was performed in the US, where regulatory agencies determine how medications are presented to physicians, and regulatory guidance represents the most common way medications are used. Nicardipine is recommended to be administered at 5 mg/hour and increased every five minutes by 2.5 mg/hour, until the target SBP range is reached or a maximum of 15 mg/hour is achieved. Once in the target SBP range, it is recommended that the infusion rate be decreased to 3 mg/hour. Labetalol dosing recommendations are for an initial IV bolus of 20 mg over two minutes, which is then repeated at 20, 40, or 80 mg injections every 10 minutes until the target SBP range is reached, or a maximum of 300 mg has been given.

Randomized patients were required to receive the first dose of study drug as soon as possible, ideally within 30 minutes of enrollment. Blood pressures were monitored via automatic cuff every five minutes during the 30 minute active phase. During the first 30 minutes after study drug initiation, the use of any additional antihypertensive was discouraged. Vital signs and potential adverse event occurrence was monitored for six hours or to ED discharge, whichever came first, after the initiation of study drug. Patient disposition (e.g., clinical decision unit, hospitalization, etc), time of transition to oral medication, and mortality status were determined at 48 hours after enrollment.

Clinical data were collected as soon as possible after enrollment, but were not required to be completed prior to study drug initiation. This included past medical history, hemoglobin, hematocrit, blood urea nitrogen (BUN), creatinine, sodium, potassium, glucose, dipstick urinalysis (and microscopic analysis if abnormal), B-type natriuretic peptide (BNP) level, and troponin. Medications taken one week prior to screening, and the assessment of baseline signs and symptoms were documented. At discharge, adverse events were recorded. Length of ICU or hospital stay, date and cause of any deaths (with autopsy data if available), were also recorded.

The presence of end-organ damage was defined as having any one of the following symptoms suggestive of a hypertensive emergency at presentation: chest pain, shortness of breath, epigastric discomfort, syncope, dizziness, blurred vision, diplopia, diminished level of consciousness, confusion, hematuria, or the development acute ischemic changes on a 12 lead electrocardiogram (ECG).

### Statistical analysis

The proportion of patients in each arm achieving the target BP within 30 minutes was compared with chi-square analysis. We predicted 55% of labetalol patients would achieve target BP range within 30 minutes. Using a two-sided alpha of 0.05, and assuming a 10% drop out rate, 113 patients in each arm provided 85% power to detect an absolute 20% effect size (75% achieving target range) in the nicardipine group.

Randomization was stratified by site. At each site patients were randomized in a 1:1 ratio. Allocation to nicardipine or labetalol was balanced in blocks of four for each of the 13 sites. Sealed envelopes were created by C5 (the coordinating research organization), and provided to the sites. Each had a label indicating the protocol name, site number, and patient study ID number. Randomization slips in the envelope contained the same information as the labels, as well as the randomized treatment. Sequence was concealed until interventions were assigned. Patients were enrolled by each site's research coordinator who was blinded to the randomization process.

Demographic tables include all randomized patients. Primary efficacy (all randomized patients) and safety (all patients receiving at least one dose of study medication) endpoints include only patients who completed the first 30 minutes of the study. For outcomes, dichotomous variables were compared by Chi-square or Fisher's Exact test where appropriate, and continuous variables by Student's T-test or appropriate nonparametric test. Missing values were not imputed and only observed values were used for analyses. A multivariable logistic model to assess the factors for "met target SBP within 30 minutes", after controlling for site differences, was developed (Table 1). All baseline variables with no more than 10% missing data points were considered for inclusion into an adjusted model. A stepwise elimination procedure was used to determine the final model. A *P *value less than 0.05 was considered as a significant risk factor and included in the final model. All statistical analyses were performed using SAS version 9.1 (SAS Institute, Cary, NC, USA).

**Table 1:** Final multivariable logistic regression model† for "met target systolic blood pressure within first 30 minutes".

Factors	Odds ratio	95% confidence interval
Nicardipine vs. labetalol	2.73	1.113-6.698
Female	3.311	1.36-8.064
No history of stroke	5.38	1.565-18.468

† Site is adjusted in the model.

## Results

**Figure 1:**
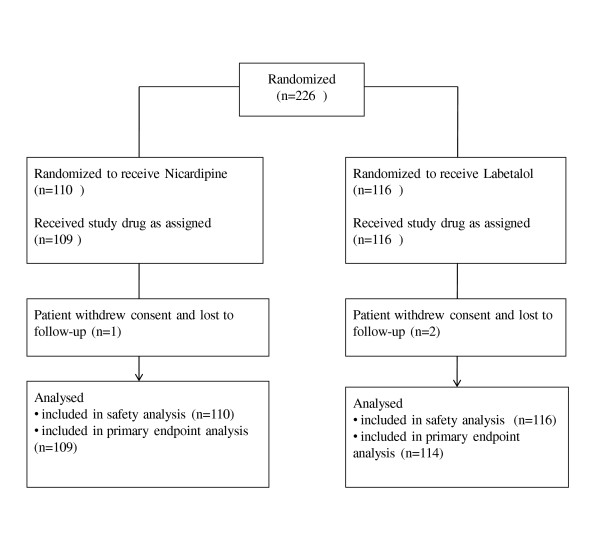
**Patient entry into CLUE trial**.

We enrolled 226 patients from 13 centers, from 16 December, 2008 until 19 January, 2010. Overall, 53% were female, and 76% were black, with a mean age of 52.6 ± 14.6 years. Randomization resulted in 110 patients receiving nicardipine and 116 labetalol, with enrollment as per Figure 1. Time from ED admission until study drug administration was similar for the nicardipine (median 2.0, interquartile range (IQR) 1.5, 2.8) and labetalol groups (median 1.9, IQR 1.3, 2.7 hours; *P *= 0.338).

Demographic, historical clinical, and laboratory parameters are presented in Table 2. Of these the nicardipine cohort was more likely to be diabetic (*P *= 0.03) or have hyperlipidemia (*P *= 0.02), and the labetalol cohort was more likely to have a social history of past and/or current smoking (*P *= 0.02; Table 2).

**Table 2:** Comparison of the characteristics of patients receiving either nicardipine or labetalol

Characteristic n (%), unless otherwise indicated	Nicardipine (*n *= 110)	Labetalol (*n *= 116)
Mean age, years ± SD	53.3 ± 15.3	51.9 ± 13.9
Female	60 (54.6)	59 (50.9)
White	28 (25.5)	24 (20.9)
African American	82 (74.6)	90 (78.3)
Smoking history	54 (49.1)	78 (67.2)
Current smoker	30 (27.3)	49 (42.2)
Stimulant use history (cocaine or amphetamines)	17 (15.5)	23 (19.8)
Median basal metabolic index (IQR)	29.3 (24.5, 34.3)	29.4 (25.1, 34.0)
Median heart rate, bpm (IQR)	85 (76, 98)	85 (73, 97)
Chest pain	33(30.0)	26 (22.4)
Diminished consciousness	1 (0.9)	5 (4.3)
Headache	48 (43.6)	58 (50.0)
Shortness of breath	34 (30.9)	27 (23.3)
Past medical history		
Hypertension	105 (96.3)	109 (94.0)
Hypertension crisis hospitalization	40 (38.8)	39 (36.4)
Hyperlipidemia	46 (43.4)	31 (27.9)
Diabetes	38 (34.6)	25 (21.7)
Coronary artery disease	17 (15.7)	15 (13.0)
Dialysis	16 (14.6)	12 (10.5)
Stroke	10 (9.2)	7 (6.2)
Heart failure	8 (7.3)	12 (10.6)
Myocardial infarction	7 (6.5)	7 (6.03)
Baseline laboratory/ECG		
Median creatinine, mg/dL (IQR)	1.2 (0.9, 3.0)	1.1 (0.9, 1.8)
Median BNP, pg/dL (IQR)	365.5 (117, 1981)	183.5 (125, 1825)
Median troponin I (ng/mL)	0.0 (0.0, 0.2)	0.0 (0.0, 0.1)
Abnormal electrocardiogram	25 (27.2)	28 (28.9)

BNP, B-type natriuretic peptide; IQR, interquartile range; SD, standard deviation.

There were no significant differences between the nicardipine and labetalol populations in regards to past medical history. More than 90% had a history of hypertension, and roughly one third had a prior hypertensive emergency hospitalization. Although there was little cardiovascular disease at presentation due to our exclusion criteria, the overall rates and interventions for these pathologies did not appear to differ between cohorts. Consistent with this similarity, there were very few differences in medications used before study enrollment. Finally, self-reported stimulant (cocaine and methamphetamine) use was similar between the two treatment groups.

Signs and symptoms of end-organ damage preceding treatment occurred in 143 (63.3%), and at similar rates between cohorts (*n *= 71, 64.5% for nicardipine, and *n *= 72, 62.1% for labetalol). The presence of end-organ damage was associated with a history of asthma, diabetes, myocardial infarction, renal failure, hepatitis, race, and a prior history of hypertension. Similar to the overall group, more end-organ damage patients receiving nicardipine were within target range within 30 minutes, than those treated with labetalol, 91.4% vs 76.1% (*P *= 0.014), respectively.

**Figure 2:**
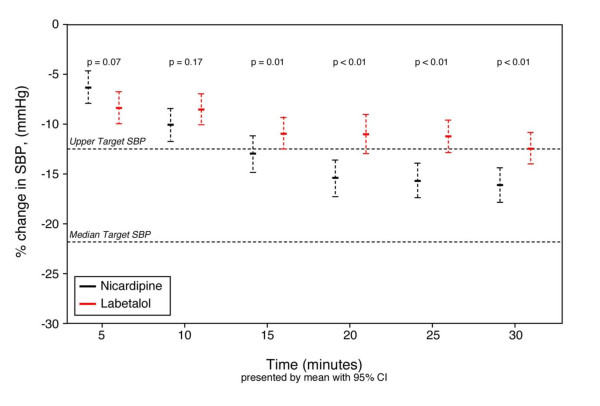
**SBP changes over time in patients randomized to receive either nicardipine or labetalol**. Mean percent change and 95% confidence interval (CI), evaluated by Student's T test, relative to presenting blood pressure, during the initial 30 minutes, with the median upper level of target range and median target range indicated by horizontal dotted lines, in patients randomized to receive either nicardipine or labetalol. SBP, systolic blood pressure.

Overall, in the intent to treat cohort the initial median SBP (IQR) was 211 (198, 226) mmHg; 212.5 (IQR 197, 230), and 212 mmHg (IQR 200, 225) in the nicardipine and labetalol groups (*P *= 0.68), respectively (Table 3). Initial target SBP were similar and are presented in Table 3 Patients treated with either nicardipine or labetalol both experienced relevant BP decreases during treatment; however, by 15 minutes the nicardipine and labetalol response curves had significantly separated (Figure 2).

**Table 3:** Initial blood pressure and target ranges at enrollment

Parameter	Descriptor	Overall *n *= 226	Nicardipine *n *= 110	Labetalol *n *= 116	*P *Value
Initial SBP (mmHg)	MEDIAN	211	212	210	0.682^NP^
	(Q1, Q3)	(198, 226)	(197, 230)	(200, 225)	
	95% CI	(210.6, 216.3)	(209.8, 218.6)	(209.0, 216.3)	
	MIN, MAX	163, 275	163, 275	180, 269	
Initial DBP (mmHg)	MEDIAN	116	112	118	0.272^NP^
	(Q1, Q3)	(105, 126)	(104, 125)	(105, 127)	
	95% CI	(114.4, 119.1)	(112.3, 119.1)	(114.5, 121.0)	
	(MIN, MAX)	(84, 195)	(84, 195)	(86, 176)	
Initial SBP target (mmHg)	MEDIAN	165	169	165	0.071 ^NP^
	(Q1, Q3)	(160, 175)	(160, 180)	(160, 170)	
	95% CI	(164.2, 168)	(165.4, 170.8)	(161.5, 166.8)	
	(MIN, MAX)	(105, 200)	(132.5, 200)	(105, 190)	

CI, confidence interval; DBP, diastolic blood pressure; np, non-parametric test; SBP, systolic blood pressure.

Within 30 minutes, nicardipine patients more often reached target range than did those treated with labetalol (91.7 vs. 82.5%, 95% confidence interval (CI) -18.0 to -0.6). Of the six BP measures obtained during the study period (BP measured every five minutes) nicardipine patients more often had five and six measures within target range than did the labetalol cohort (47.3% vs. 32.8%, 95% CI of the difference -27.2 to -1.9). To evaluate variability of BP control, the mean area under the curve (AUC) for time and depth of measures outside the SBP target range was calculated. There was no difference between nicardipine and labetalol patients with respect to median AUC (96.4 vs. 104.9 mmHg/min, *P *= 0.558). At study completion, median (IQR) SBP for the entire cohort was 165.0 (154.5, 182.0). It was 163.0 (154.0, 177.0), and 168.0 (156.0, 184.0) mmHg (95% CI of the difference between groups -13.3 to -2.0) for nicardipine and labetalol, respectively.

Important to the understanding of BP response is determining if nicardipine and labetalol dosing was appropriately aggressive. Overall, the mean (±standard deviation) number of titrations for nicardipine were 2.2 ± 1, and the mean number of doses of labetalol were 1.3 ± 0.97 (*P *< 0.001). The median (IQR) dose of nicardipine was 3.1 (2.3, 4.4) mg, compared with 40 (30, 80) mg for labetalol. The dosing ranges were 1 to 6.7 mg for nicardipine infusions, and 10 to 220 mg for bolus labetalol. Further, there were no significant differences between enrollment centers in regards to protocol deviations, time to delivery of trial drug, or duration of participation in the trial.

If patients did not attain target range SBP within the 30 minute study period, rescue medications could be given at the physician's discretion. Overall, the number of patients receiving rescue medications was not statistically different between nicardipine and labetalol groups, respectively (17 (15.5%) vs. 26 (22.4%), 95% CI of the difference -3.1 to 17.5). If nicardipine failed, the first rescue antihypertensive was most commonly labetalol (used in 11 of 17) and was not particularly effective as 47.1% required at least one more rescue medication (in addition to labetalol). If labetalol failed, the most common rescue medication was nicardipine (used in 9 of 26), and only 7.7% required additional rescue medication.

**Figure 3:**
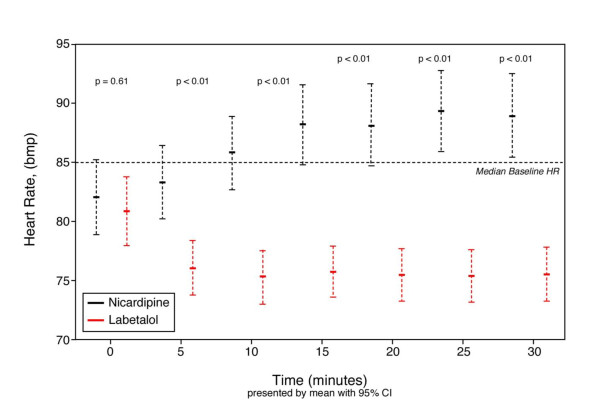
**Heart rate changes over time in patients randomized to receive either nicardipine or labetalol**. CI, confidence interval; HR, heart rate.

Adverse events attributed to study drug were rare, occurring in only one nicardipine patient who developed elevated cardiac markers after admission and no labetalol patients. Labetalol patients had slower heart rates at all time points after treatment (*P *< 0.01), although none had a heart rate below 70 (Figure 3). Only three patients did not complete the study (two labetalol and one nicardipine), due to the withdrawal of consent.

Lowering BP below target range occurred in 14 (12.7%) nicardipine, and 13 (11.2%) of the labetalol-treated patients (95% CI of the difference -10.0 to 7.1). The median (IQR) overshoot was 9.5 (3, 12.5) and 7.0 (3, 15.5) mmHg for nicardipine and labetalol cohorts, respectively (95% CI of the difference -14.3 to 7.4). The minimum and maximum overshoot of the target range were 1 and 24 mmHg for nicardipine, and 1 and 69 mmHg for labetalol.

Hours from hospital admission until ED disposition was similar between nicardipine (median 4.6, IQR 3.5, 6.6) and labetalol (median 4.6, IQR 3.1, 7.6), groups (*P *= 0.762) and at discharge from the ED or hospital, there were no differences in outpatient prescription rates, with two exceptions. As expected in a cohort with more hyperlipidemia, nicardipine patients were more likely to receive an anti-lipid agent at discharge vs. the labetalol cohort, 23.6% vs. 11.2% (95% CI of the difference -22.2 to -2.6). Nicardipine patients were also more likely to be discharged on a calcium channel blocker than were the patients treated with labetalol (38.2 vs 25.9%; 95% CI of the difference -24.4 to -0.24).

Multivariable modeling adjusting for all significant baseline variables (a total of three; drug type (*P *= 0.028), prior stroke (*P *= 0.008) and female gender (*P *= 0.008)), with enrollment site also forced into the model, showed nicardipine patients were more likely to be in target range by 30 minutes than patients receiving labetalol (odds ratio (OR) 2.73, 95% CI 1.1 to 6.7, C statistic = 0.72, Hosmer and Lemeshow goodness of fit test *P *= 0.88 showing no significant lack of fit; Table 1). Finally, to determine if chronic beta blocker or calcium channel blocker use altered treatment response, we specifically evaluated if a treatment effect occurred, based on chronic medication usage, and found no interactions.

## Discussion

Affecting nearly 500,000 patients in the US annually and contributing to about 3% of all ED visits, [2,7,8], uncontrolled hypertension can be a serious and life-threatening presentation. For those with acute end-organ dysfunction, immediate initiation of BP control is needed and, even with aggressive management, in-hospital mortality in patients presenting with acute severe hypertension exceeds 8% [9]. Achieving adequate BP control can be challenging, as each patient requires antihypertensive pharmacotherapy tailored to their specific presentation. Although candidate drugs are available for use in the acute setting, few studies have directly compared antihypertensive agents in the ED.

We conducted the first randomized comparative effectiveness trial directly evaluating the use of nicardipine and labetalol in the ED management of acute hypertension. In this we demonstrated that patients receiving nicardipine are more likely to have their BP controlled, defined as within the physician's prospectively defined target range, than patients treated with labetalol (OR 2.73, *P *= 0.0283). Furthermore, the separation of BP response curves between patients treated with either nicardipine or labetalol reached statistical significance within 15 minutes of implementation. We found only 1 in 10 nicardipine-treated patients failed to be in the target range by 30 minutes, compared with twice as many if treated with labetalol. In clinically critical conditions where rapid BP reduction would be considered optimal (e.g., intracranial hemorrhage), nicardipine may be the preferred first-line intervention.

Not unexpectedly, patients receiving labetalol had greater heart rate reduction than did the nicardipine group. Although controlled relative bradycardia is not necessarily harmful, the potential for excessive heart rate reduction may be considered, especially in light of the patient's pre-hospital medication use (e.g., already on beta-blockade), their heart rate at presentation, and if pre-existing cardiac conduction abnormalities are present. Other contraindications to beta-blockade exist, and include known chronic obstructive pulmonary disease, acute heart failure, and cocaine overdose, where the unopposed alpha effects may lead to hemodynamic instability [1]. Labetalol has some advantages over selective beta blockers as it also possesses alpha-blocking activity and may be appropriate in certain clinical situations.

Conversely, although not evaluated in this analysis, several conditions exist for which calcium channel blockers are not recommended as first-line agents. This is not necessarily because of a direct contraindication, but rather that other agents have clinical or theoretical advantages. This includes myocardial infarction, where oral beta blockers confer a mortality reduction benefit, and in the setting of aortic dissection where the negative inotropic effect of beta blockers is desirable to decrease shear forces in the false lumen of the dissection. In fact, labetalol has been specifically recommended for use in patients with aortic dissection because it has both alpha and beta blockade effects.

Overall, iatrogenic complications were rare in both treatment cohorts. Overshoot of BP below the specified range occurred in less than 15% with either nicardipine or labetalol. This may have been a function of the relatively short duration of therapy of this study, and a longer period may demonstrate different outcomes. The potential for significant overshoot represents a serious limitation in the use of any IV antihypertensive agent. Excessive overshoot, resulting in iatrogenic hypotension, may contribute to increased morbidity risk (e.g., hypoperfusion in watershed regions of the brain, resulting in acute CVA). Agents with this potential thus require close hemodynamic monitoring, which may impart an undue burden on ED nursing staff. Moreover, excessively labile anti-hypertensives have ED disposition consequences, such as requiring ICU admission rather than a step-down unit.

Our study is not without limitations. First, it was unblinded and how this may have impacted outcomes is unclear. Additionally, although most (63%) CLUE patients had symptoms consistent with a hypertensive emergency, some had elevated BP without evidence of end-organ damage. In this later group it could be argued acute BP control could have been deferred or managed with oral agents. However, our primary objective was to determine which agent was most effective at BP control for use in the ED patient population. This strictly numerical outcome requires initially hypertensive patients, but not necessarily those with end-organ injury, and makes feasible a study with much smaller numbers than would be required to power for clinical outcomes. Also, the use of this model provides valuable data to determine the most effective BP management agent while deferring the ethical conflict of treating critical patients with a potentially inferior agent.

Secondly, the separation of effect curves between nicardipine and labetalol occur after 15 minutes. This time period encompasses the period of re-dosing for labetalol, and re-dosing was at the discretion of the treating physician. Although this analysis was not designed to determine why physicians dosed agents in their chosen manner, the fact that physicians ordered fewer doses of labetalol than titrations of nicardipine may have impacted our results. Fewer labetalol titrations may be the result of the difficulty in performing frequent bolus therapy in a busy ED, or the fear of iatrogenic hypotension and bradycardia with too frequent bolus therapy. Therefore the lack of rapid BP decline in the labetalol cohort may be a result of insufficient dosing by a physician hesitant to aggressively administer successively increasing boluses of labetalol as is recommended by the FDA.

Although the six-hour observation period of our study can be criticized, this must be considered in view or our primary endpoint which was to determine which agent was most effective when rapid BP control was required. As an IV agent that requires more than six hours to control BP would have little use in the emergent scenario, we limited the time of evaluation to a period we felt was clinically relevant for rapid BP reduction.

Finally, the 30 minute definition of BP control may be questioned. However, we felt that agents requiring longer than this to control BP would be of lesser value in clinical settings where rapid BP control may be required to improve clinical outcomes. Furthermore, since the BP goals were determined by the treating ED physicians who were aware of the dosing parameters of both study drugs and the study timelines, we feel that our 30 minute goal to blood pressure control was a reasonable time limit.

We did not complete a cost analysis of the two agents. Although cost is also considered when an anti-hypertensive agent is selected, such an analysis was beyond the scope of this initial investigation.

## Conclusions

In this, the first randomized comparative effectiveness trial directly evaluating the use of a nicardipine infusion to bolus labetalol in the ED management of acute hypertension, we demonstrated that patients receiving nicardipine are more likely to have their BP controlled (OR 2.73, 95% CI 1.1 to 6.7), defined as within the physician's prospectively defined target range, than patients treated with labetalol. Although this may be the result of administration differences, this reflects actual clinical practice in how these medications are utilized. Furthermore, the need for rescue medications, or excessive BP lowering, did not appear to differ between the two cohorts. Future investigation is needed to place our findings within the context of hospital costs and resource allotment.

## Key messages

• Hypertensive emergencies require immediate, controlled BP reduction to avoid or limit end-organ damage.

• In sufficient doses, both labetalol and nicardipine lower BP.

• Patients treated with nicardipine were 2.7 times more likely to be in the target range within 30 minutes, than those treated with labetalol.

• Overshoot of BP below the specified range occurred in less than 15% of patients treated with either nicardipine or labetalol.

• Although bradycardia was more common in the labetalol group, no patient had a heart rate below 70 beats per minute.

## Abbreviations

AUC: area under the curve; min: minute; BNP: B-type natriuretic peptide; BP: blood pressure; BUN: blood, urea, nitrogen; CI: confidence interval; CLUE: Comparative effectiveness trial of IV nicardipine versus Labetalol Use in the Emergency department; CVA: cerebrovascular accident; DBP: diastolic blood pressure; ECG: electrocardiogram; ED: emergency department; FDA: Food and Drug Administration; IQR: interquartile range; IV: Intravenous; OR: odds ratio; SBP: systolic blood pressure.

## Competing interests

EKR Grant support was provided for Peacock, Varon and Baumann. There is a competing interest with The Medicine's Company involving Peacock, Varon, Baumann, Chandra and Hiestand. Co-authors Barczuk, Cannon, Cline, Diercks, Jois-Bilowich, Hsu, Kaminski, Levy, Nowak and Schrock has "no competing interest" to disclose. Chandra also has a competing interest with EKR. Hiestand has received Grant support from The Medicines Company and he has competing interest with Medtronic Inc., Biosite Inc., Inovise Medical Inc., Heartscape International, Nanosphere Inc., and Mitsubishi Medicine.

## Authors' contributions

WFP, WJ, BMB, PB, CMC, AC, DMC, DD, BH, AH, PJB, BK, PL, RMN, and JWS (all authors) were responsible for data collection and supervision of the conduct of the study. AH did the statistical analysis. WFP drafted the manuscript. WFP, WJ, BMB, PB, CMC, AC, DMC, DD, BH, AH, PJB, BK, PL, RMN, and JWS (all authors) critically reviewed the manuscript. All authors with the exception of AH contributed to the performance of this study by enrolling patients. All authors read and approved the final manuscript.
